# *Pseudomonas aeruginosa Exoprotein*-Induced Barrier Disruption Correlates With Elastase Activity and Marks Chronic Rhinosinusitis Severity

**DOI:** 10.3389/fcimb.2019.00038

**Published:** 2019-02-27

**Authors:** Jian Li, Mahnaz Ramezanpour, Stephanie A. Fong, Clare Cooksley, Jae Murphy, Masanobu Suzuki, Alkis J. Psaltis, Peter John Wormald, Sarah Vreugde

**Affiliations:** ^1^Department of Otolaryngology, The First Affiliated Hospital, Sun Yat-sen University, Guangzhou, China; ^2^Guangzhou Key Laboratory of Otorhinolaryngology, Guangzhou, China; ^3^Department of Surgery-Otolaryngology Head and Neck Surgery, University of Adelaide, Adelaide, SA, Australia; ^4^Department of Otolaryngology-Head and Neck Surgery, Hokkaido University Graduate School of Medicine, Sapporo, Japan

**Keywords:** chronic rhinosinusitis, CT-score, elastase, mucosal barrier, *Pseudomonas aeruginosa*

## Abstract

**Background:** *Pseudomonas aeruginosa* causes severe chronic respiratory diseases and is associated with recalcitrant chronic rhinosinusitis (CRS). *P. aeruginosa* exoproteins contain virulence factors and play important roles in the pathogenicity of *P. aeruginosa*, however their role in CRS pathophysiology remains unknown.

**Methods:** We isolated *P. aeruginosa* clinical isolates (CIs) and obtained clinical information from 21 CRS patients. Elastase activity of the CIs was measured at different phases of growth. Primary human nasal epithelial cells (HNECs) were cultured at air-liquid interface (ALI) and challenged with *P. aeruginosa* exoproteins or purified elastase, followed by measuring Transepithelial Electrical Resistance (TEER), permeability of FITC-dextrans, western blot, and immunofluorescence.

**Results:** 14/21 CIs had a significant increase in elastase activity in stationary phase of growth. There was a highly significant strong correlation between the *in vitro* elastase activity of *P. aeruginosa* CIs with mucosal barrier disruption evidenced by increased permeability of FITC-dextrans (*r* = 0.95, *p* = 0.0004) and decreased TEER (*r* = −0.9333, *P* < 0.01) after 4 h of challenge. Western blot showed a significant degradation of ZO-1, Occludin and β-actin in relation to the elastase activity of the exoproteins. There was a highly significant correlation between the *in vitro* elastase activity of *P. aeruginosa* CIs and CRS disease severity (for log phase, *r* = 0.5631, *p* = 0.0097; for stationary phase, *r* = 0.66, *p* = 0.0013) assessed by CT imaging of the paranasal sinuses.

**Conclusion:** Our results implicate *P. aeruginosa* exoproteins as playing a major role in the pathophysiology of *P. aeruginosa* associated CRS by severely compromising mucosal barrier structure and function.

## Introduction

*Pseudomonas aeruginosa* is an opportunistic pathogen causing a wide range of community and nosocomial infections of the airway, urinary tract, and skin such as chronic wounds and burns. It can cause aggressive infections particularly in patients with compromised host defense mechanisms or those affected by pre-existing conditions, such as cystic fibrosis (CF) (Kobayashi et al., 2009; Marvig et al., 2015). Microbiome studies indicate that *P*. *aeruginosa* dominates the lower airway niche in > 50% of adult CF patients (Filkins et al., 2012). *P. aeruginosa* secretes a number of virulence factors including proteases, toxins, phenazines, and pyocyanin that play crucial roles in infection (Coggan and Wolfgang, 2012). Secreted proteases include elastase A (LasA), elastase B (LasB), alkaline protease (AP), protease IV (PIV), and *P. aeruginosa* aminopeptidase (PAAP). They interact with the host during pathogenic infections and have a critical role in invasiveness (Janda and Bottone, 1981; Bleves et al., 2010). The expression of these virulence factors is coordinated and regulated by the las and rhl quorum sensing (QS) systems (Nouwens et al., 2003). Mutations in these systems significantly inhibit overall virulence factor production reducing the severity of both acute and chronic *P. aeruginosa* infections (Smith and Iglewski, 2003). *P. aeruginosa* elastase (LasB) is a major exoprotein and the primary elastolytic enzyme (Peters and Galloway, 1990). It plays an important role in the pathogenesis of *P. aeruginosa* infection and has been shown to have a highly efficient proteolytic activity on a range of host proteins. These include structural proteins such as elastin, critically important for maintaining the integrity of blood vessels as well as the elasticity of the lung, and collagen type III and IV, important for maintaining basement membrane integrity. *P. aeruginosa* elastase-induced disintegration of those proteins is thought to contribute to lung fibrosis and vasculitis in CF patients (Schultz and Miller, 1974; Bruce et al., 1985). LasB also degrades proteins critical in the human immune defense system such as IgA (Heck et al., 1990), IgG (Bainbridge and Fick, 1989), key components of the complement system (Schultz and Miller, 1974), and Surfactant Protein A and D (Mariencheck et al., 2003; Alcorn and Wright, 2004). Purified *P. aeruginosa* elastase has been reported to cause a transient disruption of tight junctions of airway epithelial cells and is thought to enhance the permeability of mucosal membranes facilitating tissue invasion (Azghani et al., 2000; Cowell et al., 2003; Nomura et al., 2014). Increased mucosal permeability is associated with airway inflammation and is a major contributor to respiratory infection (Coyne et al., 2002).

*P. aeruginosa* is frequently isolated from the sinonasal cavities of chronic rhinosinusitis (CRS) patients and is associated with severe recalcitrant CRS (Cleland et al., 2016). CRS patients with diabetes have been shown to be significantly more likely to have *P. aeruginosa* infections, and the infection rate of *P. aeruginosa* is correlated with the presence of nasal polyps (Zhang et al., 2014). Nasal polyps have decreased trans-epithelial electrical resistance and an irregular, decreased expression of the tight junction proteins occludin and zonulaoccludens-1 (ZO-1), critical for the normal functioning of the mucosal barrier (Soyka et al., 2012). Despite substantial evidence of the role of extracellular virulence factors and proteases including *P. aeruginosa* elastase in airway pathologies, the correlation between *P. aeruginosa* elastase activity of CRS clinical isolates and disease severity in the context of CRS remains unknown.

This study determined the *P. aeruginosa* elastase activity in clinical isolates (CIs) harvested from the sinonasal cavities of CRS patients, and determined the relationship between elastase activity, effect on the mucosal barrier structure and function, and its relation to objective CRS disease severity scores.

## Materials and Methods

### Study Participants and Disease Characteristics

This study was carried out in accordance with the recommendations of The Queen Elizabeth Hospital Human Research Ethics Committee with written informed consent from all subjects in accordance with the Declaration of Helsinki. HNECs for *in vitro* experiments were harvested from 4 independent control patients without CRS or cystic fibrosis (CF) (two males, two females, aged 32–71 years). Demographics, diagnoses, Lund-Mackay Computed Tomography (CT) scores, Lund-Kennedy endoscopic scores, Sino-nasal Outcome Test 22 (SNOT-22) scores (within 12 months of the swab being taken) and other clinical information of CRS patients with *P. aeruginosa* infection were collected.

### Human Nasal Epithelial Cell Culture

HNECs were harvested from the inferior turbinate using a nasal brush and cultured as described (Ramezanpour et al., 2016, 2017). Briefly, extracted HNECs were suspended in Bronchial Epithelial Growth Media (BEGM, CC-3170, Lonza, Walkersville, MD, USA), supplemented with 2% Ultroser G (Pall Corporation, Port Washington, NY, USA). The cell suspension was depleted of monocytes using anti-CD68 (Dako, Glostrup, Denmark) coated culture dishes, and HNECs expanded in routine cell culture conditions of 37°C humidified air with 5% CO_2_ in collagen coated flasks (Thermo Scientific, Walthman, MA, USA). HNECs were tested at passage two and confirmed to be of epithelial lineage via reactivity to pan-Cytokeratin and CD45 antibodies (both from Abcam, Cambridge, MA, USA), and a Diff-Quick staining method used in the assessment of cell morphology by professional cytologists (IMVS, The Queen Elizabeth Hospital, Woodville, Australia).

### Air Liquid Interface Culture

HNECs were maintained at an Air-Liquid Interface (ALI), following the Lonza ALI culture method (Lonza, Walkersville, USA). Briefly, Transwells (BD Biosciences, San Jose, California, USA) were coated with collagen (Stem cell Technologies, Australia). 0.7 × 10^6^ HNECs were seeded in 100 μL B-ALI growth medium into the apical chamber of the Transwell plate and 500 μL of B-ALI growth medium was added to the basal chamber in all wells containing the inserts. Cells were incubated at 37°C. On day 3 after seeding, B-ALI growth medium was removed from the apical and basal chambers and 500 μL B-ALI differentiation medium was added to the basal chamber only, exposing the apical cell surface to the atmosphere.

### Preparation of *P. aeruginosa* Exoproteins

*P. aeruginosa* PAO1 reference strain was obtained from American Type Culture Collection (ATCC) and in total 21 *P. aeruginosa* clinical isolates were harvested from the sinonasal cavities of CRS patients using endoscopically-guided sinus swabs. *P. aeruginosa* CIs were isolated by an independent pathology laboratory (Adelaide Pathology Partners, Adelaide, South Australia) and multi-locus sequence typing (MLST) was used to confirm taxonomic classification of *P. aeruginosa* (Fong et al., 2017). Growth curves were established for each strain to ascertain the log and stationary phases of planktonic bacterial growth in Luria Broth (LB) medium, grown shaking at 180 rpm at 37°C as described (Balda et al., 1993). Exoproteins of all strains were collected at log phase and stationary phase, sterilized by 0.22 μm filters and concentrated using Vivaspin protein concentrator spin columns with 3000 molecular weight cut-off (MWCO) (GE Healthcare Bio-Sciences, Pittsburgh, USA). Concentration of exoproteins was measured using NanoOrange™ Protein Quantitation Kit (Thermo Scientific, Walthman, MA, USA) according to manufacturer's instructions.

### Trans Epithelial Electrical Resistance (TEER) Analysis

An EVOM voltohmmeter (World Precision Instruments, Sarasota, FL, USA) was used to measure TEER as described (Ramezanpour et al., 2016, 2017). Briefly, 100 μL of B-ALI growth medium was added to the apical chamber of ALI cultures to form an electrical circuit across the cell monolayer and into the basal chamber. Only wells displaying baseline resistance readings greater than 500 Ω/cm^2^ were used for the experiments. Exoproteins (50 μL exoproteins in LB medium and 50 μL B-ALI growth medium), purified *P. aeruginosa* elastase (0.5, 5, 25, and 50 μg/mL in B-ALI growth medium) (Sigma-Aldrich, St. Louis, MO, USA) and negative control (50 μL B-ALI growth medium/50 μL LB medium for exoprotein experiments and B-ALI growth medium for experiments with purified elastase) were added to the apical chamber of each well, and TEER measurements were obtained at time 0, 1, 2, 3, and 4 h for exoproteins and at time 0 and 24 h for the purified *P. aeruginosa* elastase. TEER values were normalized to the average TEER measurement prior to challenge. Between samples the probe was washed stepwise in 70% ethanol, PBS, then B-ALI medium to minimize cross-contamination between samples. Each well was measured 3 times via two ports on either side of the insert.

### Dextran-FITC Permeability Assay

Paracellular permeability was studied by measuring the apical-to-basolateral flux of Dextran-FITC-4 kDa (Sigma-Aldrich). Briefly, after treating the cells for 4 h (exoproteins) or 24 h (purified *P. aeruginosa* elastase), the upper chambers were filled with 3 mg/mL of Dextran-FITC and incubated at 37°C for 2 h. Forty Microliter samples were recovered from the bottom chamber and serially diluted in a 96-well plate (Sigma-Aldrich), and the fluorescence was measured with a microplate fluorometer (FLUOstar Optima, BMG Labtech, Ortenberg, Germany).

### Cell Cytotoxicity Assay

Exoproteins were collected from basal chambers of each sample following TEER measurements and the release of lactate dehydrogenase (LDH) was measured by using Cytotox Homogeneous Membrane Integrity Assay (Promega, Australia) as described (Ramezanpour et al., 2016).

### *Pseudomonas aeruginosa* Elastase Activity Analysis

The elastase activity of all *P. aeruginosa* strains was determined using Elastin-Congo red (ECR; Sigma-Aldrich). Briefly, for each sample, 100 μL concentrated filter-sterilized bacterial exoprotein was added to 900 μL of ECR buffer (100 mM Tris-HCl, pH 7.5, 1 mM CaCl2, 20 mg ECR) and incubated for 18 h at 37°C with agitation at 200 rpm. Then insoluble ECR was removed by centrifugation at 16,000 g for 15 min at room temperature, and the absorbance was measured at 495 nm. Fresh LB medium was used as the blank control, and LB with ECR as the negative control. Secreted elastase activity was expressed as the ratio of the OD495 and OD600 absorbance value.

### Immunofluorescence Staining

HNEC monolayers were fixed with 2.5% formalin in phosphate-buffered saline (PBS) for 15 min, washed three times with cold PBS and permeabilized in 100 μL 0.1% Triton X-100 in PBS. Cells were rinsed with Tris-buffered saline-0.5% Tween (TBST) four times and blocked with serum free blocker (SFB; Dako, Glostrup, Denmark) for 60 min, at room temperature. Mouse monoclonal anti-human ZO-1, diluted to 10 μg/mL in TBST-10% SFB, or Rabbit polyclonal anti-human Occludin, diluted to 2 μg/mL in TBST-10% SFB (both Invitrogen, Carlsbad, CA, USA), was added to the monolayers and allowed to incubate overnight at 4°C. Cells were washed 3 times with TBST followed by incubation with 2 μg/mL anti-mouse Cy3 or anti-rabbit Alexa-488 conjugated secondary antibody (Jackson ImmunoResearchLabs Inc., West Grove, PA, USA) for 1 h at room temperature. Cells were rinsed in TBST followed by adding 200 ng/mL of 4′,6-diamidino-2-phenylindole (DAPI, Sigma-Aldrich). Membranes were rinsed once with ultrapure water and 95% ice-cold ethanol was added for 1 h at 4°C. Membranes were transferred to a glass slide and a drop of anti-fade mounting medium (Dako) was added before cover-slipping. Samples were visualized using a LSM700 confocal laser scanning microscope (Zeiss Microscopy, Germany).

### Western Blot of Tight Junction and Cytoskeleton Proteins

HNECs in 6-well plates were treated for 24 h with 5% (v/v) exoproteins from 2 strains at log phase (log) and stationary phase (sta). Luria-Bertani (LB) medium 5% (v/v) and B-ALI growth medium were used as negative controls. Western analysis was performed using the X Cell Sure Lock Mini Cell System M (Invitrogen) as previously described (Clark et al., 2011). Membranes were probed with Mouse monoclonal anti-human ZO-1 (1:125 dilution, Invitrogen), Rabbit polyclonal anti-human Occludin (3 μg/mL, Invitrogen) and mouse monoclonal anti-human β-actin antibodies (1:1,000; Sigma–Aldrich). Equal protein loading (10 μg) was assessed using the rabbit polyclonal anti-human Profilin-1 antibody (1:5,000 dilution; Invitrogen). Goat anti-rabbit IgG horseradish peroxidase (HRP) (Abcam) and Goat anti-mouse IgG HRP (Abnova, Taipei, Taiwan, China) were used as secondary antibodies. Luminescence was detected using the ImageQuant LAS-4000 biomolecular imager (Tokyo, Japan), and densitometry analyses performed using Multi-Gauge software (V3.0, Fugifilm Science Lab, Tokyo, Japan). Log-transformed density scores were normalized to both profilin-1 and the biological control.

### Statistical Analysis

Data are presented as mean ± Standard Error of Mean (SEM). The TEER, permeability assay, cell cytotoxicity assay experiments were performed using 4 biological replicates from 3 independent donors. Elastase activity was measured 3 times in 3 independent experiments. Statistical analysis of all data was carried out using ANOVA, followed by Tukey honest significant difference (HSD) *post-hoc* test. The Spearman correlation analysis was performed between two variables. Spearman's rank correlation coefficient and *P-*value were calculated to describe the correlation result. These tests were performed using SPSS software (version 18) and GraphPad Prism 5 (version 5.01).

## Results

### *Pseudomonas aeruginosa* Exoproteins Reduce the Transepithelial Electrical Resistance (TEER) of HNEC-ALI Cultures

**Figure 1:**
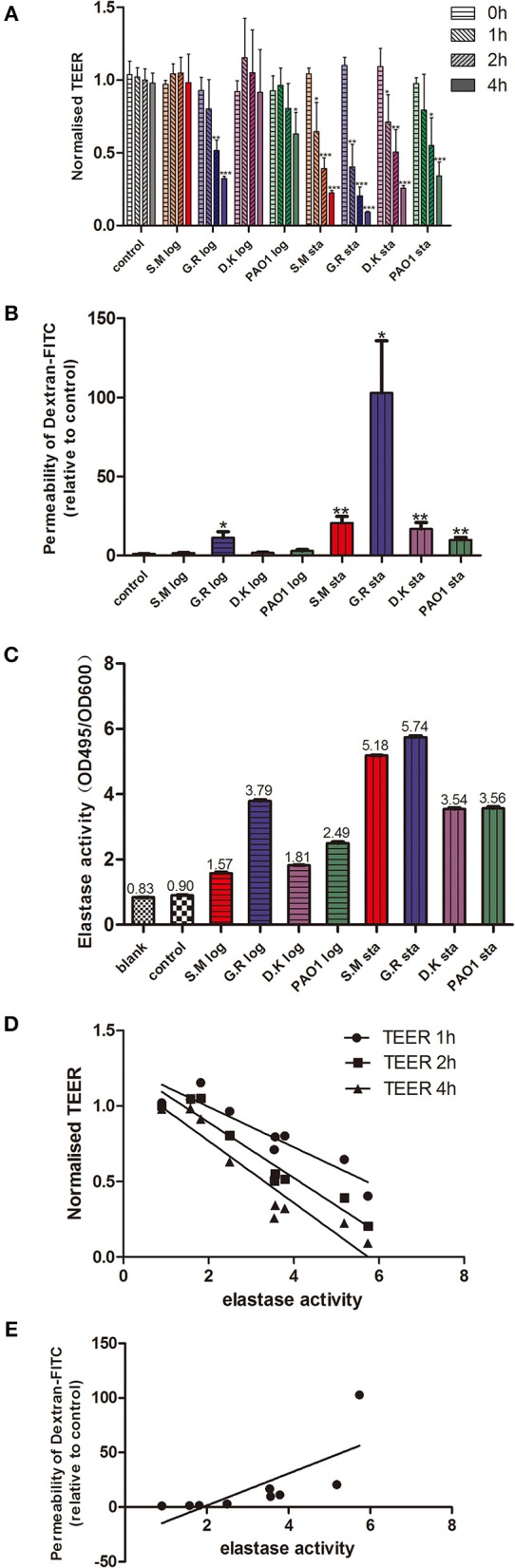
*P. aeruginosa* exoproteins cause mucosal barrier dysfunction in correlation with elastase activity. *P. aeruginosa* exoproteins collected from 3 clinical isolates (S.M.-red color in Figures 1A–C; G.R.-blue color in Figures 1A–C; D.K.-purple color in Figures 1A–C) and 1 reference strain (PAO1, green color in Figures 1A–C), at exponential phase (log) and stationary phase (sta) of growth were applied to HNEC-ALI cultures, followed by measuring Transepithelial Electrical Resistance (TEER) hourly for 4 h **(A)** and passage of Dextran-FITC 4 h after application **(B)**. The elastase activity was measured by Elastin-Congo red (ECR) reaction and shown as OD495/OD600 ratio. Fresh LB medium was used as blank control (blank), and LB with ECR as a negative control (control) **(C)**. The Spearman's rank correlation coefficient was determined between the elastase activity and TEER at different time points **(D)** or Dextran-FITC permeability **(E)**. The values are shown as mean±SEM for *n* = 4. ^*^p < 0.05, ^**^p < 0.01, ^***^p < 0.001. ANOVA, followed by Tukey HSD *post-hoc* test.

*P. aeruginosa* exoproteins contain virulence factors including secreted proteases that interact with the host during pathogenic infections and have a critical role in invasiveness (Janda and Bottone, 1981; Bleves et al., 2010). To test the effect of exoproteins on the mucosal barrier *in vitro*, primary HNEC-ALI cultures were established from 4 independent control patients without CRS or CF (two males, two females, aged 32–71 years) followed by measuring Trans Epithelial Electrical Resistance (TEER) and passage of Dextran-FITC. Exoproteins were collected from log and stationary growth phases of 4 *P. aeruginosa* strains [PAO1 reference strain and three CIs collected from the sinus cavities of CRS patients—one with CF (S.M.), two without], filtered and protein concentration measured (Supplementary Figure 1 and Supplementary Table 1). TEER was examined after application of exoproteins at different time points. Whilst only some exoproteins from log phase cultures affected TEER values after 2 h (one isolate) and 4 h (two isolates) of challenge, stationary phase exoproteins of all 4 strains significantly reduced the TEER values at 1 to 2 h after application. The stationary phase exoproteins of the G.R strain had the largest effect on TEER values, with a highly significant reduction of TEER after 1 h of application (2.73-fold reduction compared to the medium control, *P* = 0.0292). This further decreased to a 12.04-fold reduction after 4 h of application (*P* < 0.0001). Also, exponential phase exoproteins of the G.R strain significantly reduced TEER within 2 h of application (Figure 1A).

### *Pseudomonas aeruginosa* Exoproteins Increase the Paracellular Permeability of HNEC-ALI Cultures

TEER measures the flux of all ions across the epithelium and cannot be used to measure tight junction size (Shen et al., 2011). To assess this, we measured the flux of Dextran-FITC tracers across HNEC-ALI cultures. Exoproteins from all stationary phase cultures as well as from the CI G.R log phase caused significant enhancement of paracellular permeability within 4 h after application. Stationary phase exoproteins from the G.R strain had the strongest effect on permeability with a >100-fold increase of Dextran-FITC crossing the HNECs monolayer 6 h after application (Figure 1B).

### Nasal Epithelial Layer Disruption Does Not Correlate With Cytotoxicity

To assess the contribution of cellular toxicity on the increased permeability of the HNEC-ALI cultures, the effect of *P. aeruginosa* exoproteins on cellular toxicity was determined by measuring LDH release from HNECs 4 h after application. There was no statistically significant change in LDH release after stimulation with *P. aeruginosa* exoproteins compared to control (Supplementary Figure 1B).

### Purified *P. aeruginosa* Elastase Decreases HNEC Barrier Function in a Dose-Dependent Way

*P. aeruginosa* elastase plays an important role in the pathogenesis and invasion of *P. aeruginosa* infection and can cause a transient disruption of tight junctions of airway epithelial cells (Azghani et al., 2000; Cowell et al., 2003; Nomura et al., 2014). However, the effect of *P. aeruginosa* elastase on primary HNECs is not known. Application of purified *P. aeruginosa* elastase to HNEC-ALI cultures had a dose-dependent effect on mucosal barrier function with a significant reduction in TEER observed for 25 μg/mL (*P* = 0.0149) and 50 μg/mL (*P* = 0.0004). An increased paracellular permeability of Dextran-FITC was seen after application of 50 μg/mL purified *P. aeruginosa* elastase 24 h after application (*P* < 0.0001) (Supplementary Figure 2).

### Elastase Activity Differs Amongst Clinical *Pseudomonas aeruginosa* Isolates

Given that clinical isolates might differ in their virulence factor production and activation, we next wanted to determine the elastase activity of different strains at different phases of growth. The *P. aeruginosa* elastase activity was measured by ECR reaction and expressed as the ratio of the OD495 and OD600 absorbance values. Different strains had different elastase activities for both exponential and stationary phase of growth. For all strains tested, the elastase activity from stationary phase was higher than from exponential phase (*p* < 0.0001). The highest elastase activity was from the G.R strain (Figure 1C).

### Elastase Activity Significantly Correlates With Monolayer Permeability and TEER Values of *Pseudomonas aeruginosa* Clinical Isolates *in vitro*

To assess a potential relationship between the mucosal barrier function and elastase activity, the Spearman's rank correlation coefficient was determined between the elastase activity and TEER at different time points or Dextran-FITC permeability, respectively. There was a significant very strong positive correlation between elastase activity and Dextran-FITC permeability (*r* = 0.9500, *p* < 0.01) and a significant strong negative correlation between elastase activity and TEER after 1 h (*r* = −0.8667, *P* < 0.01), 2 h (*r* = -0.8833, *P* < 0.01) and 4 h (*r* = −0.9333, *P* < 0.01) challenge with *P. aeruginosa* exoproteins (Figures 1D,E and Table 1).

**Table 1:** Spearman correlation analysis of elastase activity with Dextran-FITC permeability and TEER at different time-points.

**Correlation analysis of elastase activity**	**Dextran-FITC permeability**	**TEER 1 h**	**TEER 2 h**	**TEER 4 h**
Spearman r	0.9500	−0.8667	−0.8833	−0.9333
*P-*value (two-tailed)	0.0004	0.0045	0.0031	0.0007
*P*-value summary	^***^	^**^	^**^	^***^

*There was a positive correlation between elastase activity and Dextran-FITC permeability and negative correlations between elastase activity and TEER (1, 2, and 4 h after treatment with P. aeruginosa exoproteins)*.

*** *P < 0.001*,

** *P < 0.01*.

### *P. aeruginosa* Exoproteins Cause Disruption and Degradation of Tight Junction Proteins and Cytoskeleton

**Figure 2:**
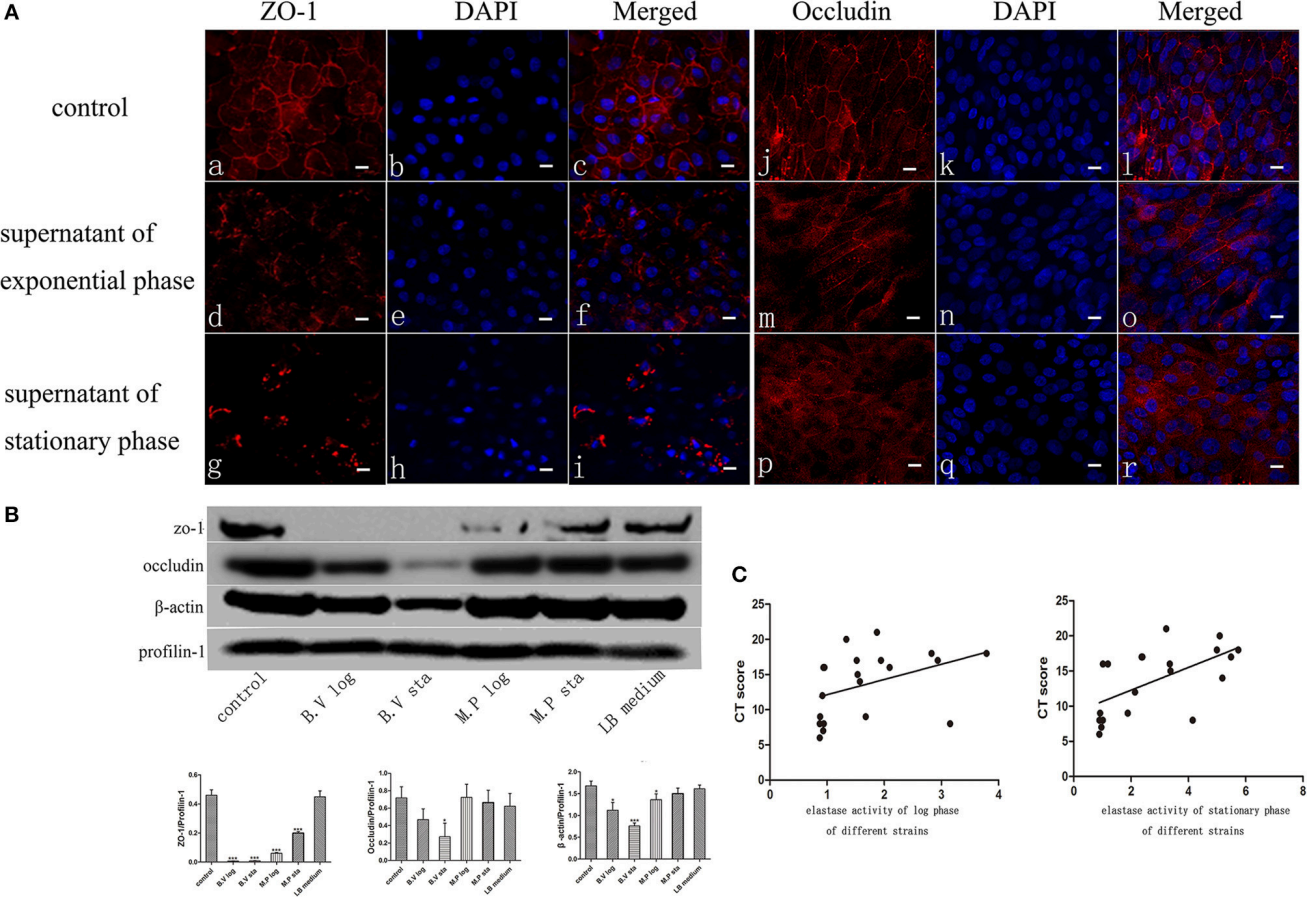
*P. aeruginosa* exoproteins cause disruption of ZO-1, Occludin and Actin of primary human nasal epithelial cells (HNECs). **(A)** The effect of *P. aeruginosa* B.V strain exoproteins at Log phase (d-f and m-o) and Stationary phase (g-i and p-r) on the localization of Zona Occludens-1 (ZO-1) (red staining with DAPI stain in blue in a-i) and Occludin (red staining with DAPI stain in blue in j-r) using immunofluorescence confocal laser scanning microscopy 6 h after application of the exoproteins. **(B)** Western blot analysis and relative densitometric quantification of Zona Occludens-1 (ZO-1), Occludin and β-actin after application of 5% (v/v) exoproteins of *P. aeruginosa* strains B.V and M.P for 24 h. HNEC monolayers without treatment were control group. Luria-Bertani (LB) medium was used as the negative control. Profilin-1 was used as the loading control. Densitometric quantification relative to Profilin-1. **(C)** Spearman correlation analysis of Lund-Mackay Computed Tomography (CT) scores with elastase activity of 20 *P. aeruginosa* clinical isolates at log phase or stationary phase of growth. Results are representative of three independent experiments. ^*^p < 0.05, ^***^p < 0.001. ANOVA, followed by Tukey HSD *post-hoc* test.

The Apical Junctional Complex (AJC) contains tight and adherens junctions and maintains mucosal barrier integrity. To get insights into the potential mechanism of increased mucosal barrier permeability induced by application of the exoproteins, the localization of Zona Occludens-1 (ZO-1) and Occludin was examined by immunofluorescence staining and confocal laser scanning microscopy 6 h after application of *P. aeruginosa* exoproteins. In control cells, ZO-1 and Occludin were consistently located at the periphery of epithelial cells. ZO-1 and Occludin discontinuous immunolocalization was seen after challenge with *P. aeruginosa* exoproteins, particularly those from stationary phase cultures (Figure 2A). To investigate whether *P. aeruginosa* exoproteins affected ZO-1, Occludin and β-actin protein levels, western blots were performed after application of exoproteins from 2 CIs at log and stationary growth phase, and in relation to the elastase activity of those isolates. The exoproteins of the B.V strain had high elastase activity (mean elastase activity, 5.86 of B.V stationary phase, 2.93 of B.V log phase) and caused a complete degradation of ZO-1 (compared to control, *P* < 0.0001 for B.V log and B.V sta), a significant degradation of Occludin (compared to control, *P* = 0.0187 for B.V sta, *P* = 0.0713 for B.V log) and significant reduction in β-actin protein levels (compared to control, *P* = 0.0103 for B.V log, *P* = 0.0003 for B.V sta). The exoprotein of the M.P strain had a mean elastase activity of 2.17 for the stationary phase and 0.92 for the log phase, and also caused significant degradation of ZO-1 (compared to control, *P* < 0.0001 for M.P log, *P* = 0.0003 for M.P sta) and significantly decreased the protein level of β-actin (compared to control, *P* = 0.0338 for M.P log, *P* = 0.1427 for M.P sta) (Figure 2B).

### Elastase Activity of *P. aeruginosa* Clinical Isolates Significantly Correlates With Disease Severity CT Scores of CRS Patients

To elucidate if elastase activity of CIs collected from the nasal cavity of CRS patients correlates with CRS disease severity, we randomly selected a further 18 *P. aeruginosa* clinical isolates. Phylogenetic analysis using MLST analysis was performed on 12 CIs and showed no significant relationship between the isolates (Supplementary Table 2) (Fong et al., 2017). Growth curves were established (Supplementary Figure 3A), exoproteins collected at log phase (12–13 h) and stationary phase (24–25 h) (see Supplementary Table 2 for OD values), filtered with Vivaspin 2 (3000 MWCO), followed by determination of protein concentration and elastase activity by ECR reaction. There was no significant increase in protein concentration between log phase and stationary phase of the same isolates. 10/18 and 11/18 isolates significantly increased their elastase activity compared to control in the Log and Stationary phase, respectively (Supplementary Figures 3B,C). There was a significant negative correlation between the elastase activity and exoprotein concentration of log phase and stationary phase cultures (*r* = –0.66, *p* < 0.0008 and *r* = –0.57, *p* < 0.005) (Supplementary Table 3, Supplementary Figure 4). Lund-Mackay Computed Tomography (CT) scores, Lund-Kennedy endoscopic scores, the 22-item Sinonasal Outcome Test (SNOT-22) score (all within 12 months of the swab being taken), and number of previous sinus surgeries were used to estimate CRS disease severity. Demographics and disease severity scores are shown in Supplementary Table 2. Spearman correlation analysis showed there was no correlation between elastase activity and subjective evaluation scores (VAS and SNOT-22 scores), Lund-Kennedy endoscopic score, or number of previous sinus surgeries (data not shown). However, there was a strong significant correlation between Lund-Mackay CT scores and elastase activity of the isolates at log phase (*r* = 0.5631, *P* < 0.01) and stationary phase (*r* = 0.6689, *P* < 0.01) (Figure 2C, Table 2).

**Table 2:** Spearman correlation analysis of Lund-Mackay Computed Tomography (CT) scores with elastase activity at log phase or stationary phase.

**Correlation analysis of CT score**	**Elastase activity of log phase**	**Elastase activity of stationary phase**
Spearman r	0.5631	0.6689
*P-*value (two-tailed)	0.0097	0.0013
*P-*value summary	^**^	^**^

*20 clinical isolates from 20 patients with CT scores and clinical information were included in the analysis. There was a positive correlation between CT scores and elastase activity of P. aeruginosa*.

** *P < 0.01*.

## Discussion

Our study showed a differential ability of *P. aeruginosa* clinical isolates exoproteins to disrupt mucosal barrier structure and function in a manner that directly correlated with the elastase activity of those isolates. Strikingly, *in vitro P. aeruginosa* elastase activity strongly correlated with CT-scan scores of disease severity in patients with CRS. In addition, dose-dependent detrimental effects on the HNEC-ALI mucosal barrier function were observed after application of purified *P. aeruginosa* elastase. Our findings contribute significant insights into the pathophysiology of CRS and implicate *P. aeruginosa* elastase or exoproteins secreted in conjunction with elastase as correlating with *P. aeruginosa-*associated CRS disease severity. Not only does it support the notion that barrier dysfunction may play a key role in chronic inflammation and polyp formation in CRS patients, it implicates *P. aeruginosa* exoproteins as major contributing factors in that process implicating these exoproteins as potentially important therapeutic targets.

Whilst our results indicate elastase as potentially directly contributing to the mucosal barrier disruption observed, it is known that the secretion of *P. aeruginosa* elastase is a highly coordinated process regulated by the las and rhl quorum sensing (QS) systems regulating the expression of over 50 genes (Whiteley et al., 1999; Nouwens et al., 2003). Further studies are required to elucidate the exact role and contribution of elastase in this process, by specifically blocking the *P. aeruginosa* elastase activity and/or constructing and characterizing *P. aeruginosa LasB* and *LasA* mutants.

In this study, 61% (14/20) of the *P. aeruginosa* clinical isolates harvested from within the sinonasal cavities demonstrated significant elastase activity. This is low given that 100% of clinical *P. aeruginosa* isolates are reported to carry the *LasB* gene and can produce elastase (Finnan et al., 2004). Whilst the molecular basis for this finding remains to be defined, mutations in *LasR*, the transcriptional regulator of the *LasB* gene, have been shown to reduce *P. aeruginosa* elastase production (Pearson et al., 1997) and are associated with lung function decline and altered sensitivity to antibiotics in patients with CF (Hoffman et al., 2009).

Nomura et al has previously shown that purified *P. aeruginosa* elastase could disrupt the epithelial barrier in a transient manner (Nomura et al., 2014), however the effect was slower and less profound compared to the effects observed in this study. This could be due to reduced functional properties of the recombinant purified elastase and/or differences in concentration used. Also, Nomura et al. used HNECs transfected with the human catalytic subunit of the telomerase reverse transcriptase (hTERT) and it is unclear how that might affect the elastase-induced effect on the barrier.

Details of the mechanistic effects of *P. aeruginosa* exoproteins against the epithelial barrier remain to be explored but this study indicates an extensive disruption of both cytoskeleton and apical junctional complex (AJC) proteins. This might indicate a disruption of common gene regulatory elements and/or signal transduction pathways as has previously been demonstrated for *P. aeruginosa* elastase (Nomura et al., 2014). The AJC comprises tight and adherens junctions and maintains epithelial cell polarity and mucosal barrier integrity. The AJC includes the transmembrane proteins ZO-1, occludin, Junction Adhesion Molecule 1 (JAM-A), claudin family members, and linker proteins that bind the underlying actin cytoskeleton. Disruption and re-organization of AJC proteins is a hallmark of mucosal barrier dysfunction. It is frequently found in severe chronic inflammatory diseases of the gut, skin, and airway such as inflammatory bowel disease, allergic dermatitis, and asthma (Weidinger et al., 2008; Turner, 2009). In the context of CRS, bacterial secreted products as well as different cytokines have been reported to disrupt the mucosal barrier *in vitro*, and barrier dysfunction has been proposed to have a critical role in the pathogenesis of this disease (Tieu et al., 2009; Soyka et al., 2012; Malik et al., 2015; Ramezanpour et al., 2016; Murphy et al., 2017). Results of previous studies with primary alveolar pneumocytes indicated that *P. aeruginosa* elastase enhanced epithelial permeability by redistribution and disruption of tight junction proteins (Azghani et al., 2000). Likewise, using HNECs, our results indicated that *P. aeruginosa* secreted products induced a severe disruption of the barrier function and degraded the TJ and cytoskeleton proteins ZO-1, occludin and β-actin. Strikingly, the extent of barrier dysfunction strongly correlated with the elastase activity of those products. *P. aeruginosa* elastase has been shown to have a highly efficient proteolytic activity on a range of host proteins, facilitating tissue invasion and contributing to airway pathology such as lung fibrosis and vasculitis in cystic fibrosis patients (Schultz and Miller, 1974; Bruce et al., 1985). *P. aeruginosa* elastase also contributes to *P. aeruginos*a's ability to evade and disarm the host immune system by degrading proteins of the human immune defense system (Schultz and Miller, 1974; Bainbridge and Fick, 1989; Heck et al., 1990; Mariencheck et al., 2003; Alcorn and Wright, 2004). Taken together, the concerted action of exoproteins such as *P. aeruginosa* elastase in increasing mucosal permeability and degrading innate immune defense mechanisms could lead to an ongoing, but ineffective, immune response, which contributes to the severe inflammation and polyp formation in *P. aeruginosa*-associated CRS patients.

## Author Contributions

All authors agree to be accountable for all aspects of the work and gave their final approval of the version to be published. In addition, JL, MR, SF, and CC had substantial contributions to the acquisition and analysis of data and drafting the work. JM, MS, AP, and PW had substantial contributions to the design of the work and critical revision. SV had substantial contribution to the conception and design of the work, drafting of the work and critical revision.

### Conflict of Interest Statement

The authors declare that the research was conducted in the absence of any commercial or financial relationships that could be construed as a potential conflict of interest.

## Supplementary Material

Supplementary Figure 1 Growth curves of *P. aeruginosa* and Lactate Dehyrogenase (LDH) assays. Growth curves from 3 *P. aeruginosa* clinical isolates (S.M., G.R., D.K.) and from PAO1 were established and exoproteins collected at 11–12 h (exponential phase, log) and 22–23 h (stationary phase, sta) of growth **(A)** followed by application to HNEC-ALI cultures and measuring cell viability by LDH assay 4 h after stimulation, calculated relative to control HNECs **(B)**. The values are shown as mean ± SEM for *n* = 4. ANOVA, followed by Tukey HSD *post hoc* test.

Supplementary Figure 2 Purified *P. aeruginosa* elastase disrupts the barrier function of HNECs. Different concentrations of purified *P. aeruginosa* elastase (0.5, 5, 25, and 50 μg/mL in B-ALI medium) were added onto HNEC-ALI cultures for 24 h followed by measuring TEER **(A)** and paracellular permeability **(B)**. B-ALI medium was used as control. The values are shown as mean ± SEM for *n* = 4. ^*^*p* < 0.05, ^**^*p* < 0.01, ^***^*p* < 0.001. ANOVA, followed by Tukey HSD *post hoc* test.

Supplementary Figure 3 Growth curves and elastase activity of 18 *P. aeruginosa* CRS clinical isolates. Cell number was estimated by measuring OD600 and exoproteins collected at 12–13 h as exponential phase and 24–25 h as stationary phase **(A)**. Elastase activity of log phase **(B)** and stationary phase **(C)** was shown as OD495/OD600 ratio. The values are shown as mean ± SEM for *n* = 3. Acronyms refer to different strains of clinical isolates. Fresh LB medium was used as the blank control, and LB with ECR as the negative control.

Supplementary Figure 4 Spearman correlation analysis of elastase activity with exoprotein concentration of log phase and stationary phase cultures of 22 strains. Twenty-one clinical isolates from 21 patients and 1 PAO1 reference strain was included in the analysis. There were negative correlations between elastase activity and exoprotein concentration of log phase and stationary phase cultures.

Supplementary Table 1 Elastase activity and protein concentrations for PAO1 and clinical isolates.

Supplementary Table 2 Demographics, disease severity scores, elastase activity and multi-locus sequence typing (MLST) analysis of *P. aeruginosa* clinical isolates.

Supplementary Table 3 Spearman correlation analysis of elastase activity with exoprotein concentration of log phase and stationary phase of 22 strains. There were negative correlations between elastase activity and exoprotein concentration of log phase and stationary phase. ^***^*P* < 0.001, ^**^*P* < 0.01.
