# Supplementary figures and images for: Pseudomonas aeruginosa Exoprotein-Induced Barrier Disruption Correlates With Elastase Activity and Marks Chronic Rhinosinusitis Severity
Source: Front Cell Infect Microbiol. 2019 Feb 27;9:38. doi: 10.3389/fcimb.2019.00038 (PMC6400838; doi:10.3389/fcimb.2019.00038)

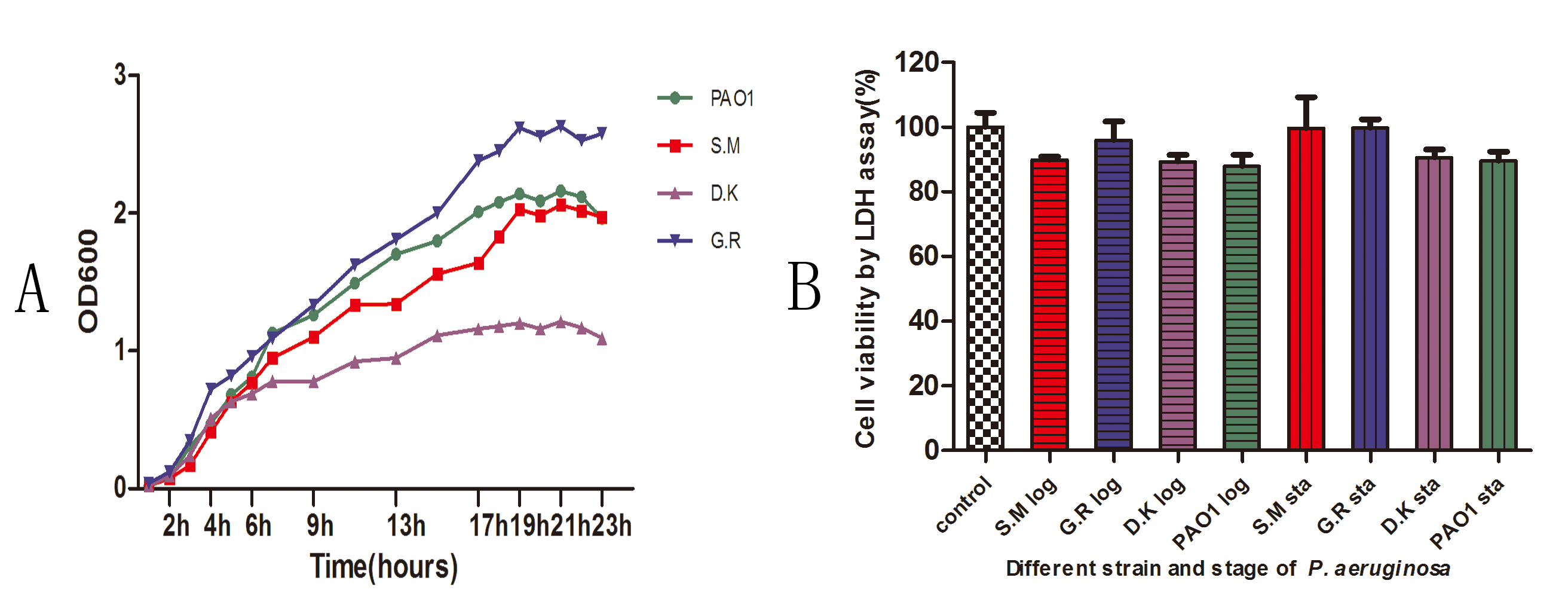

Supplement: Supplementary Figure 1 — Growth curves of P. aeruginosa and Lactate Dehyrogenase (LDH) assays. Growth curves from 3 P. aeruginosa clinical isolates (S.M., G.R., D.K.) and from PAO1 were established and exoproteins collected at 11–12 h (exponential phase, log) and 22–23 h (stationary phase, sta) of growth (A) followed by application to HNEC-ALI cultures and measuring cell viability by LDH assay 4 h after stimulation, calculated relative to control HNECs (B). The values are shown as mean ± SEM for n = 4. ANOVA, followed by Tukey HSD post hoc test. [file Image_1.TIF]

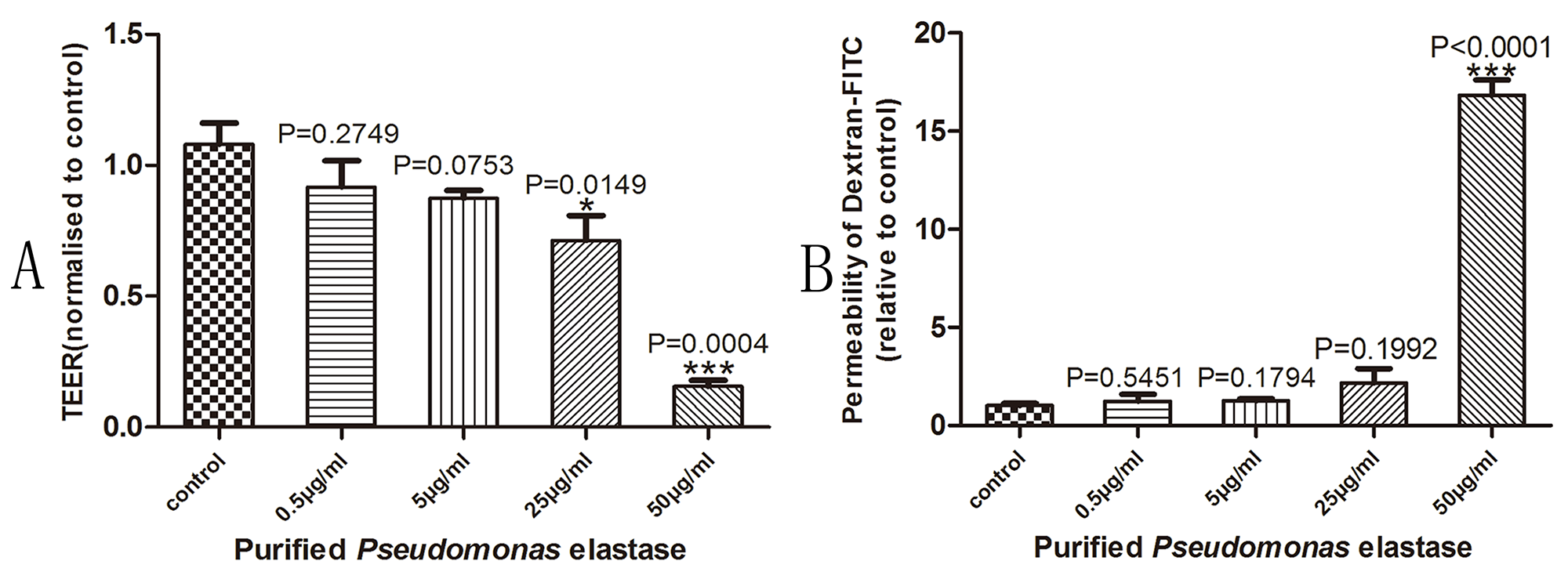

Supplement: Supplementary Figure 2 — Purified P. aeruginosa elastase disrupts the barrier function of HNECs. Different concentrations of purified P. aeruginosa elastase (0.5, 5, 25, and 50 μg/mL in B-ALI medium) were added onto HNEC-ALI cultures for 24 h followed by measuring TEER (A) and paracellular permeability (B). B-ALI medium was used as control. The values are shown as mean ± SEM for n = 4. *p < 0.05, **p < 0.01, ***p < 0.001. ANOVA, followed by Tukey HSD post hoc test. [file Image_2.TIF]

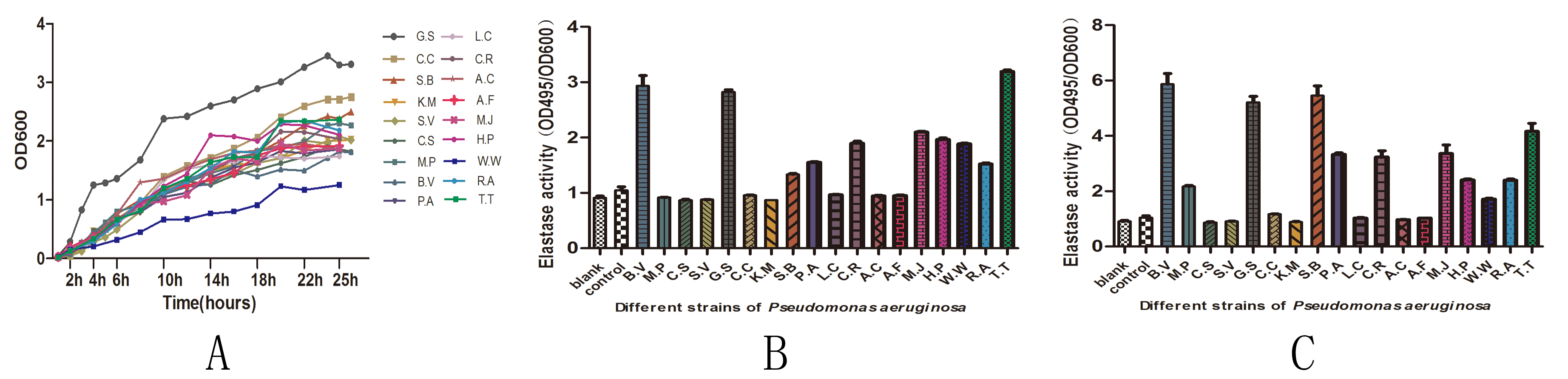

Supplement: Supplementary Figure 3 — Growth curves and elastase activity of 18 P. aeruginosa CRS clinical isolates. Cell number was estimated by measuring OD600 and exoproteins collected at 12–13 h as exponential phase and 24–25 h as stationary phase (A). Elastase activity of log phase (B) and stationary phase (C) was shown as OD495/OD600 ratio. The values are shown as mean ± SEM for n = 3. Acronyms refer to different strains of clinical isolates. Fresh LB medium was used as the blank control, and LB with ECR as the negative control. [file Image_3.TIF]

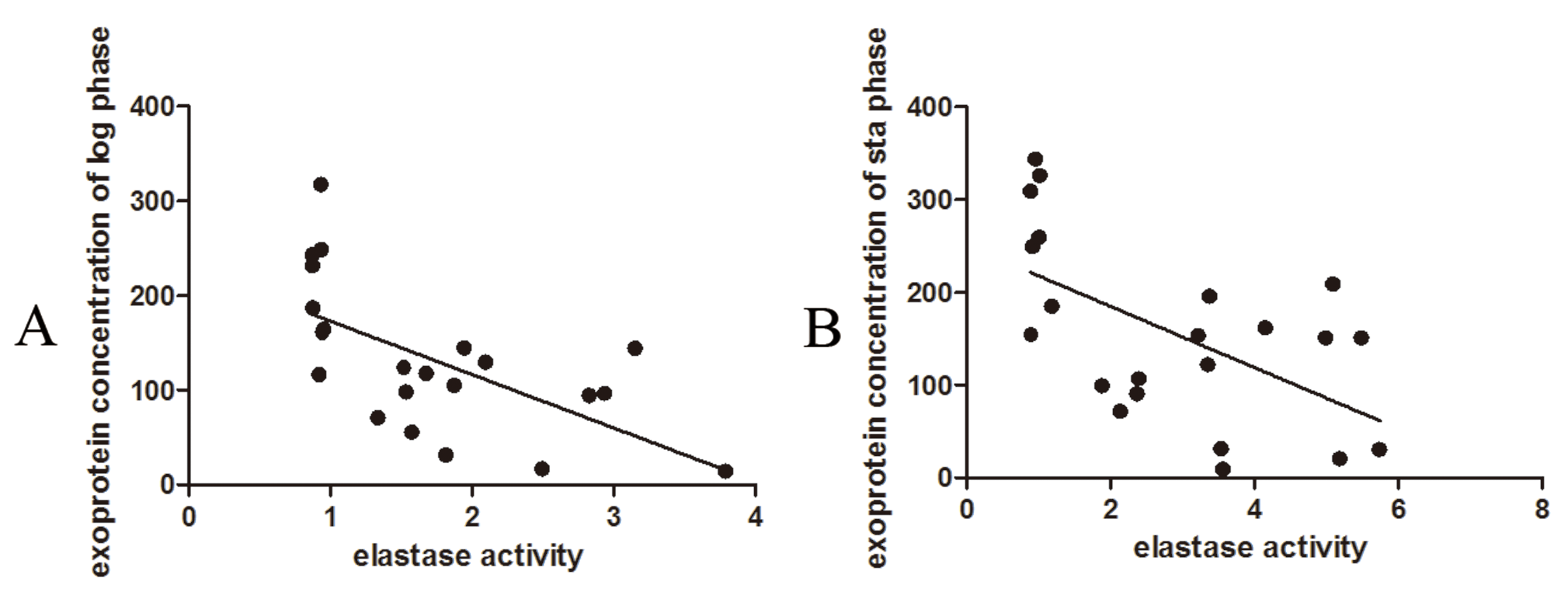

Supplement: Supplementary Figure 4 — Spearman correlation analysis of elastase activity with exoprotein concentration of log phase and stationary phase cultures of 22 strains. Twenty-one clinical isolates from 21 patients and 1 PAO1 reference strain was included in the analysis. There were negative correlations between elastase activity and exoprotein concentration of log phase and stationary phase cultures. [file Image_4.TIF]
