# Supplementary material for: Pseudomonas aeruginosa Exoprotein-Induced Barrier Disruption Correlates With Elastase Activity and Marks Chronic Rhinosinusitis Severity
Source: Front Cell Infect Microbiol. 2019 Feb 27;9:38. doi: 10.3389/fcimb.2019.00038 (PMC6400838; doi:10.3389/fcimb.2019.00038)
Supplement: Supplementary Table 1 — Elastase activity and protein concentrations for PAO1 and clinical isolates. [file Table_1.DOCX]

**Table S1: Elastase activity and protein concentrations for PAO1 and Clinical Isolates**

| **Strains** | **Elastase activity of log phase** | **Exoprotein concentration of log phase (ug/ml)** | **Elastase activity of stationary phase** | **Exoprotein concentration of stationary phase (ug/ml)** |
| --- | --- | --- | --- | --- |
| G.R | 3.789203 | 14.6025 | 5.739496 | 30.3425 |
| S.M | 1.574534 | 55.4145 | 5.183673 | 20.5255 |
| D.K | 1.813814 | 31.347 | 3.541026 | 31.056 |
| B.V | 2.937173 | 96.4485 | 5.486957 | 151.0535 |
| M.P | 0.9181287 | 116.3455 | 2.133333 | 71.4455 |
| C.S | 0.871345 | 242.7855 | 0.8888889 | 309.343 |
| S.V | 0.8786127 | 186.749 | 0.9166667 | 249.3675 |
| G.S | 2.827027 | 94.2175 | 4.995193 | 150.728 |
| C.C | 0.9545454 | 164.5375 | 1.189349 | 184.6725 |
| K.M | 0.8711656 | 231.4475 | 0.8974359 | 154.379 |
| S.B | 1.337278 | 71.224 | 5.093137 | 208.6955 |
| P.A | 1.535135 | 97.8615 | 3.374331 | 195.734 |
| L.C | 0.9444444 | 161.203 | 1.012739 | 259.51 |
| C.R | 1.871658 | 105.025 | 3.222222 | 153.52 |
| A.C | 0.9318182 | 317.0825 | 0.9602649 | 344.277 |
| A.F | 0.9371428 | 248.348 | 1.013514 | 326.294 |
| M.J | 2.09375 | 129.522 | 3.35 | 121.9985 |
| H.P | 1.942308 | 144.569 | 2.394737 | 107.325 |
| W.W | 1.677596 | 117.3975 | 1.882353 | 99.572 |
| R.A | 1.51948 | 124.312 | 2.365979 | 90.435 |
| T.T | 3.151659 | 143.8305 | 4.147368 | 161.807 |
| PAO1 | 2.49435 | 16.4245 | 3.5625 | 9.0255 |
