# Supplementary material for: Pseudomonas aeruginosa Exoprotein-Induced Barrier Disruption Correlates With Elastase Activity and Marks Chronic Rhinosinusitis Severity
Source: Front Cell Infect Microbiol. 2019 Feb 27;9:38. doi: 10.3389/fcimb.2019.00038 (PMC6400838; doi:10.3389/fcimb.2019.00038)
Supplement: Supplementary Table 2 — Demographics, disease severity scores, elastase activity and multi-locus sequence typing (MLST) analysis of P. aeruginosa clinical isolates. [file Table_2.docx]

**Supplementary Table S2. Demographics, disease severity scores, elastase activity and multi-locus sequence typing (MLST) analysis of *P. aeruginosa* clinical isolates.**

| Patients Clinical isolates | Gender | Age  (years) | Main diagnosis | Other diagnoses | Operation times | Lund-Mackay score | Lund-Kennedy score | SNOT-22 | Elastase activity (stationary phase, OD495/OD600) | OD600 values at log phase | OD600 values at stationary phase | MLST sequence type |
| --- | --- | --- | --- | --- | --- | --- | --- | --- | --- | --- | --- | --- |
| B.V | M | 55 | CRSwNP | Diabetes | 4 | 17 | 10 | 36 | 5.861±0.388 | 1.193±0.093 | 1.780±0.061 |  |
| G.R | F | 76 | CRSsNP | Asthma | 7 | 18 | 12 | 60 | 5.740±0.046 | 1.830±0.161 | 2.581±0.049 | 910 |
| S.B | M | 56 | CRSwNP | Asthma | 6 | 20 | 6 | 55 | 5.448±0.357 | 1.303±0.166 | 2.433±0.061 | 1517 |
| G.S | M | 64 | CRSwNP | Diabetes | 2 | 18 | 12 | 58 | 5.211±0.211 | 2.467±0.117 | 3.353±0.084 |  |
| S.M | F | 44 | CRSwNP | CF | 1 | 14 | 6 | # | 5.184±0.007 | 1.411±0.129 | 2.017±0.045 | 499 |
| T.T | F | 47 | CRSsNP |  | 1 | 8 | 12 | # | 4.162±0.291 | 1.397±0.227 | 2.353±0.015 |  |
| D.K | F | 29 | CRSwNP |  | 2 | # | # | 69 | 3.541±0.034 | 0.996±0.101 | 1.157±0.061 | 1399 |
| M.J | F | 84 | CRSsNP |  | # | 16 | # | # | 3.359±0.312 | 1.190±0.291 | 1.890±0.062 |  |
| P.A | M | 44 | CRSwNP |  | 1 | 15 | 4 | # | 3.331±0.055 | 1.183±0.174 | 1.827±0.035 | 446 |
| C.R | F | 80 | CRSwNP | Asthma | 5 | 21 | 9 | # | 3.234±0.220 | 1.337±0.150 | 2.117±0.067 | 242 |
| H.P | F | 68 | CRSwNP | Asthma | 9 | 17 | 12 | 31 | 2.407±0.028 | 1.590±0.454 | 2.223±0.099 | 270 |
| R.A | M | 74 | CRSwNP |  | 11 | 17 | 20 | 29 | 2.399±0.035 | 1.323±0.210 | 2.283±0.091 | 399 |
| M.P | F | 70 | CRSsNP | Asthma | 5 | 12 | 4 | 17 | 2.171±0.038 | 1.291±0.184 | 2.293±0.040 | 274 |
| W.W | F | 63 | CRSwNP |  | 3 | 9 | 4 | # | 1.712±0.030 | 0.700±0.061 | 1.217±0.042 | 988 |
| C.C | F | 62 | CRSwNP | Asthma,  Diabetes | 9 | 16 | 14 | 107 | 1.171±0.015 | 1.577±0.145 | 2.723±0.023 |  |
| L.C | F | 66 | CRSsNP |  | # | 8 | # | # | 1.033±0.013 | 1.487±0.131 | 1.723±0.015 |  |
| A.F | M | 78 | CRSsNP |  | 3 | 16 | 12 | # | 1.027±0.004 | 1.251±0.096 | 1.903±0.021 |  |
| A.C | F | 52 | CRSsNP | Asthma | 2 | 7 | 4 | 23 | 0.967±0.008 | 1.530±0.165 | 1.910±0.053 |  |
| S.V | F | 87 | CRSwNP |  | 4 | 9 | 20 | 38 | 0.908±0.010 | 1.232±0.106 | 2.020±0.060 | 155 |
| K.M | F | 76 | CRSwNP |  | 2 | 8 | 6 | 10 | 0.885±0.017 | 1.333±0.155 | 2.017±0.012 | 395 |
| C.S | F | 48 | CRSsNP |  | 2 | 6 | 14 | 62 | 0.872±0.015 | 1.227±0.107 | 1.870±0.056 |  |

CRSsNP= chronic rhinosinusitis without nasal polyps

CRSwNP= chronic rhinosinusitis with nasal polyps
