# Supplementary material for: Pseudomonas aeruginosa Exoprotein-Induced Barrier Disruption Correlates With Elastase Activity and Marks Chronic Rhinosinusitis Severity
Source: Front Cell Infect Microbiol. 2019 Feb 27;9:38. doi: 10.3389/fcimb.2019.00038 (PMC6400838; doi:10.3389/fcimb.2019.00038)
Supplement: Supplementary Table 3 — Spearman correlation analysis of elastase activity with exoprotein concentration of log phase and stationary phase of 22 strains. There were negative correlations between elastase activity and exoprotein concentration of log phase and stationary phase. ***P < 0.001, **P < 0.01. [file Table_3.DOCX]

| Correlation analysis of elastase activity | Exoprotein concentration of log phase | Exoprotein concentration of stationary phase |
| --- | --- | --- |
| Spearman r | -0.6601 | -0.5731 |
| P value (two-tailed) | 0.0008 | 0.0053 |
| P value summary | *** | *** |

**Supplementary Table 3. Spearman correlation analysis of elastase activity with exoprotein concentration of log phase and stationary phase of 22 strains.** There were negative correlations between elastase activity and exoprotein concentration of log phase and stationary phase. *** P<0.001, ** P<0.01.
